# Edward Jenner Museum

**DOI:** 10.3201/eid1704.101680

**Published:** 2011-04

**Authors:** William Foege

**Affiliations:** Author affiliation: Bill & Melinda Gates Foundation, Seattle, Washington, USA

**Keywords:** viruses, vaccines, Edward Jenner, smallpox, vaccination, cowpox, history of medicine, museum review, Another Dimension

Ground zero for vaccinology is Edward Jenner’s home in Berkeley, England. Here Edward Jenner worked, studied, and practiced as a country doctor—and later dominated a decade of my life! Jenner knew that poets talked about the nice complexions of milkmaids, and he heard a milkmaid say she was immune to smallpox because she had acquired cowpox. He came to believe in the protective effects of cowpox after careful review of the experiences of milkmaids during smallpox outbreaks. He spent a dozen years observing before he experimented with the transfer of cowpox from a lesion on the hand of Sarah Nelms, a milkmaid, to 8-year-old James Phipps, the son of a local laborer, in May 1796. Although Jenner had no concept of viruses, immune systems, or vaccinology, he used science to help imitate what he saw happening in nature.

**Figure 1:**
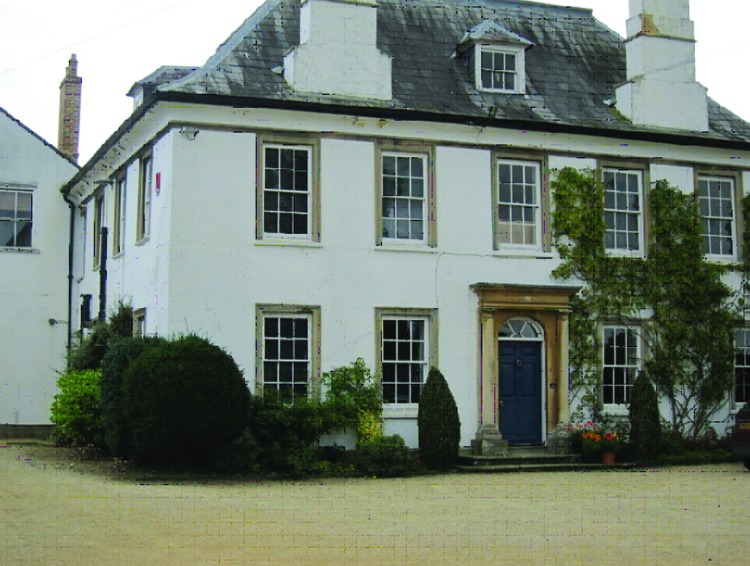
The Edward Jenner home, Edward Jenner Museum, Berkeley, Gloucestershire, England. Photo credit: William Foege.

The Jenner home (Figure 1) in Berkeley has served as a museum (www.jennermuseum.com) for the past 25 years, and with both facts and artifacts continues to tell the story of Jenner’s life and discoveries. In September 2010, Sarah Parker, the museum’s director, provided us with a day of her time to show what has been done, to discuss what is planned, and to answer questions about the museum and Dr. Jenner. The only part of the house off limits to our group was Jenner’s study, which had to be viewed through a glass partition but was fully visible. While he was sitting at this desk in 1823, writing up notes from a house call to verify the death of the coroner, Jenner had a stroke. He was then carried upstairs to his bedroom and never regained consciousness. On one wall is a bookcase that is standing open as it was at the time of his death.

**Figure 2:**
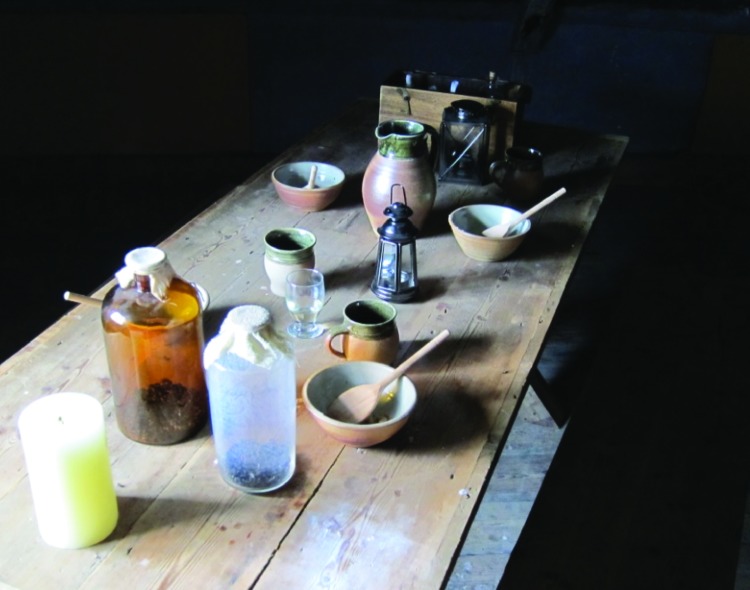
Work table, Edward Jenner Museum, Berkeley, Gloucestershire, England. Photo credit: William Foege.

The home provides a fascinating view into the life of a village doctor in the early 19th century. I was struck by a work table set up as it would have been at that time. The light through the window produced shadows that made an intriguing picture (Figure 2), which would have been spoiled by the use of a flash.

The building is a maze of stairways and doors. Although Jenner’s medical office was actually down the street from the house, in addition to that office, he had work areas at home where he studied a variety of natural phenomena. One irony of Jenner’s life is that he was admitted to the Royal Society not on the basis of his smallpox discoveries, but because of his observations that cuckoos laid their eggs in other birds’ nests.

At one point Sarah Parker asked if we had any idea regarding the identity of a fan-shaped object standing upright in front of a fireplace. I said I didn’t know, of course, but it looked like the scapula of a whale. She looked dejected, because that was actually the correct answer. Evidently, at some point, a whale had beached near Berkeley, and Jenner, with his usual curiosity, dissected it, keeping the scapula for his personal collection.

We even explored the attic, which contains materials from 200 years ago that have not yet been catalogued or studied. We viewed, through windows, the water collection system used for the house. The first two floors of the house were used by Jenner and his family while servants lived in the attic. This provided another fascinating insight to a social dynamic that was common then but is difficult to imagine now.

**Figure 3:**
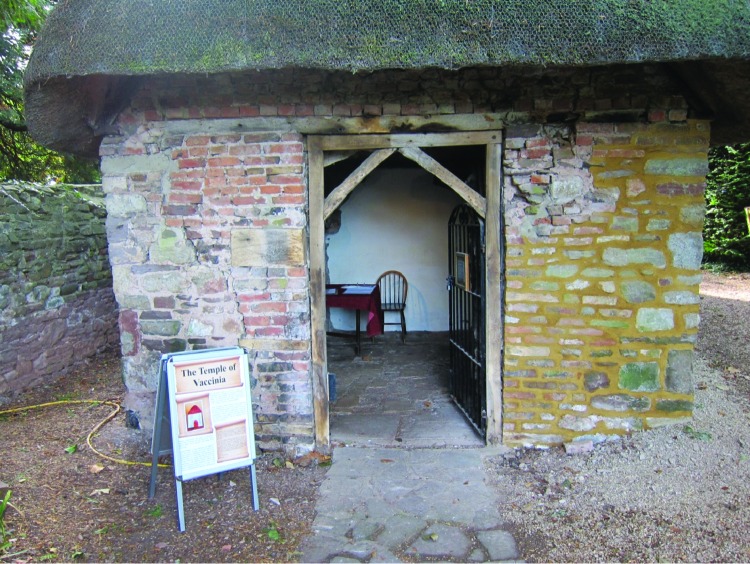
Cottage used by Edward Jenner for vaccinating children, Edward Jenner Museum, Berkeley, Gloucestershire, England. Photo credit: William Foege.

His garden was a joy to observe. At one end of the garden is the thatched roofed house (Figure 3) where poor children would assemble on 1 day each week, and Jenner would vaccinate them at no cost. On the other side are the grape vines that he planted. The vines are unusual looking because the roots grow outside but the stems are then passed into a greenhouse, which assists in the maturation and the collection of the grapes. We were allowed to eat grapes from these vines.

Next door to the house is a church with a burial ground containing the graves of many of the people who figure into Jenner’s story. Twenty-six years ago, before the museum opened, my son Michael and I stopped in this churchyard on our return from a trip to India. We were fortunate to encounter a vicar who was retired but interested in Jenner. He had returned to conduct a funeral, and we met him by chance. He spent 3 hours recounting the history of Jenner and the community of Berkeley.

While standing in the church talking with us, the vicar noticed a woman quietly waiting for him to finish. He asked if he could help her, and she said she was looking for the grave of an ancestor, Sarah Nelms. I asked, “You mean the milkmaid?” She was as surprised by my question as I was at hearing the name.

Also close by is Berkeley Castle. It has been inhabited by the Berkeley family since it was first built in the twelfth century and is said to be the oldest castle in the United Kingdom inhabited continuously by the same family. Jenner’s need to tend the Berkeley family is believed to have interrupted his smallpox work, and this obligation could account for the 2-year delay in publishing his paper after the vaccination of James Phipps.

By great fortune, we had the chance to meet Professor Gareth Williams, internist, expert on diabetes and obesity, and a medical college dean and author. He lives within biking distance of the museum and had decided to use a sabbatical to write a book on Jenner and smallpox (Angel of death: the story of smallpox. Basingstoke [UK]: Palgrave-MacMillan; 2010). His interest stemmed from noticing the Jenner name in the history of his church. Jenner’s father and 2 brothers were clergymen. Professor Williams also has a reputation for baking cakes for various occasions, and he appeared at the museum with a cake for our meeting!

The museum continues to expand but runs on a shoestring budget. (I say this for the benefit of anyone who has ideas on funding.) Ms. Parker has developed many displays in the past few years and has plans for the future, all depending on resources. She is even considering restoring part of the building so it can serve as a bed-and-breakfast inn for people who might want to spend a night in Jenner’s house. In addition, the recently formed Edward Jenner Society (www.edwardjennersociety.org), which is dedicated to vaccinology, uses the Jenner home for its meetings.

**Figure 4:**
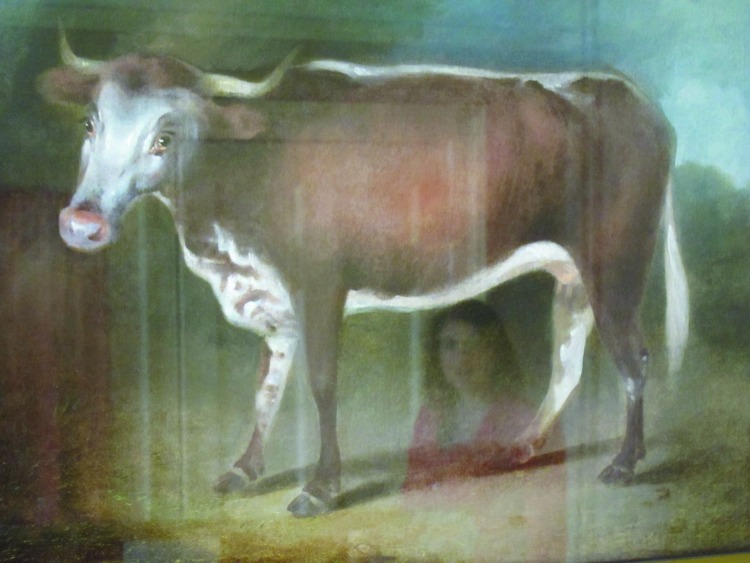
Painting of a cow, Edward Jenner Museum, Berkeley, Gloucestershire, England. Photo credit: William Foege.

There is a legend regarding ghosts in the building, and a photograph is displayed in the house that supposedly shows a ghost in the background. Scientists, of course, do not give much credence to such stories. I did try to take a picture of a painting of the cow that had infected Sarah Nelms with the cowpox virus, which in turn was transferred to James Phipps. The first picture had too much glare, so I took a second one less than a minute later. I was totally surprised to see the image of a woman directly below the cow’s stomach (Figure 4). Because we know of no pictures of Sarah Nelms, the milkmaid involved, it would be tempting to believe it was she. But we have to make some assumptions on the identity of the person in the second photo!

Much more could be told about the insights to be gained from this museum, but instead I recommend a visit. Although it is a natural place to visit if one is interested in vaccines, I firmly believe that it is also ground zero for public health. The modern public health movement can be dated to May 14, 1796, when a vaccine for smallpox became available.

